# Early Enteral Feeding in Preterm Infants: A Narrative Review of the Nutritional, Metabolic, and Developmental Benefits

**DOI:** 10.3390/nu13072289

**Published:** 2021-07-01

**Authors:** Melissa Thoene, Ann Anderson-Berry

**Affiliations:** Department of Pediatrics, College of Medicine, University of Nebraska Medical Center, Omaha, NE 68198-1205, USA; alanders@unmc.edu

**Keywords:** enteral, feeding, preterm, nutrition, growth, development

## Abstract

Enteral feeding is the preferred method of nutrient provision for preterm infants. Though parenteral nutrition remains an alternative to provide critical nutrition after preterm delivery, the literature suggests that enteral feeding still confers significant nutritional and non-nutritional benefits. Therefore, the purpose of this narrative review is to summarize health and clinical benefits of early enteral feeding within the first month of life in preterm infants. Likewise, this review also proposes methods to improve enteral delivery in clinical care, including a proposal for decision-making of initiation and advancement of enteral feeding. An extensive literature review assessed enteral studies in preterm infants with subsequent outcomes. The findings support the early initiation and advancement of enteral feeding impact preterm infant health by enhancing micronutrient delivery, promoting intestinal development and maturation, stimulating microbiome development, reducing inflammation, and enhancing brain growth and neurodevelopment. Clinicians must consider these short- and long-term implications when caring for preterm infants.

## 1. Introduction

Appropriate nutrition and growth are critical therapies for the preterm infant population. Multiple studies have concluded that early and higher nutrient provision influences growth and clinical outcomes in preterm infants, such as neurodevelopment, prevention of bronchopulmonary dysplasia (BPD), or decreased risk of retinopathy of prematurity (ROP) [1,2,3,4,5]. A growth-related study by Ehrenkranz et al. explicitly quantified that increasing quartiles for weight gain for extremely low birth weight infants was associated with improved neurodevelopmental outcomes at 18 months of age [1]. Early postnatal nutrition delivery must be prioritized, as increased energy and protein intakes solely during the first week of life have been associated with long-term neurodevelopment [2]. However, when considering most optimal sources of nutrition for preterm infants, the provision of mother’s own milk is the recommended choice [6]. Multiple benefits have been described, with examples including enhanced immunity and a reduced risk of necrotizing enterocolitis (NEC), BPD, late onset sepsis, and ROP in preterm infants [7,8,9]. While the literature strongly supports provision of adequate nutrition, there is evidence that additional benefit is conferred when enterally provided compared to parenterally. Therefore, the purpose of this narrative review is to summarize benefits of enteral feeding in preterm infants (<37 weeks gestation) during the first one month of life (Table 1).

## 2. Review of the Literature

As this was not a systematic review or meta-analysis, literature was reviewed as available and applicable to early life enteral feeding in preterm infants. Though recently published articles were preferable, less recent publications were included, if relevant. Literature results are summarized as a narrative review.

### 2.1. Enteral Nutrient Provision

Early enteral feeding provides vital nutrients and promotes necessary growth in preterm infants. Though priority may often be on adequacy of total energy and protein provision for these infants, adequate delivery of micronutrients (including 13 vitamins and multiple minerals) also remains essential to promote optimal physical growth and neurodevelopment [36]. Early delivery of enteral feeding may markedly increase overall provision of essential micronutrients via fortification or nutrient supplementation compared to parenteral nutrition alone, especially if parenteral additive shortages exist [37]. In addition, parenteral micronutrient formulations may not provide sufficient amounts for preterm infants receiving total parenteral nutrition. In the example of vitamin D, joint guidelines for parenteral provision are 200–1000 International Units (IU) per day (or 80–400 IU/kilogram(kg)/day) [38]. However, recommendations may not be met by extremely low birth weight infants who only receive 120 IU/day based on dosing instructions for standard parenteral multivitamin formulations [37]. Consequently, enteral provision becomes the most feasible alternative to enhance delivery, with human milk fortification able to be initiated at enteral volumes as low as 20 milliliters/kg/day (mL/kg/day) or at first feeding [32,33], though the addition of such will increase enteral feeding osmolality [35]. Higher collective provisions of multiple micronutrients (e.g., calcium, phosphorus, vitamin D) via early initiation and attainment of full enteral feeding theoretically supports appropriate postnatal growth [28], such as bone mineralization. Given that 80% of bone mineralization occurs in the third trimester of fetal development [39], optimizing the provision of these nutrients in the first one month of life is crucial, as this accounts for one third of this vital period.

Benefits conferred to preterm infants by providing mother’s own milk compared to preterm infant formula or heat-processed donor human milk has been analyzed [7,40,41,42]. However, beyond these considerations, early enteral feeding also allows for the supplementation of essential nutrients not standardly added to parenteral nutrition formulations. One example is iron, which is associated with improved hematological status and may have implications related to neurodevelopment in preterm infants [43,44]. Though the age of starting supplementation may vary, initiation at 2 weeks of life may reduce the need for transfusion and later iron-deficiency anemia [45,46]. Thus, neonates receiving minimal enteral feeding in the first month of life will not receive early benefit from this critical nutrient.

Further benefit of early enteral feeding is provision of nutrients not deemed essential for life but having potential to impact health. Carotenoids are one example of non-essential micronutrients found in human milk or infant formula, including lutein which concentrates in brain and eye tissue at levels dependent on dietary intake [47,48]. An enteral supplementation trial of lutein in infants born <33 weeks gestation (*n* = 203) reported that among infants with retinopathy, fewer supplemented infants progressed to severe stages (8 vs. 28%) [49]. As this nutrient is not included or available as a parenteral nutrition additive, enteral feeding becomes the only method of delivery, with provision increasing parallel to higher volume of enteral intake. Furthermore, these nutrients may have cumulative effects on retinal development [50], so the most benefit will be attained by early initiation and progressive advancement of enteral feeding after preterm birth.

### 2.2. Gastrointestinal Development

**Figure 1:**
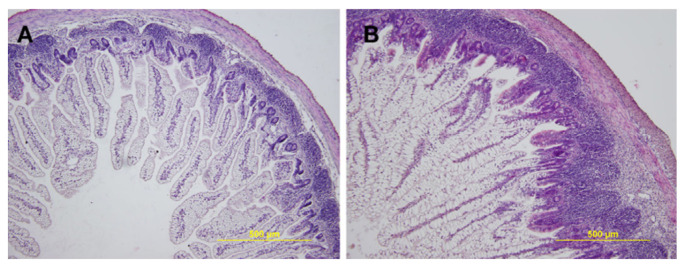
Cross Section Stain of Villi in the Ileum of Preterm (**A**) vs. Term (**B**) Piglets. Figure reproduced from Frontiers in *Immunology*, Vol. 9, Page 5 as available via Open Access and under terms of the Creative Commons Attribution License. Original article published as “Preterm Life in Sterile Conditions: A Study on Preterm, Germ-Free Piglets” by Splichalova et al., 2018 [51].

Preterm infants are born with incomplete organ development, including the intestinal tract, as evidenced in Figure 1.

**Figure 2:**
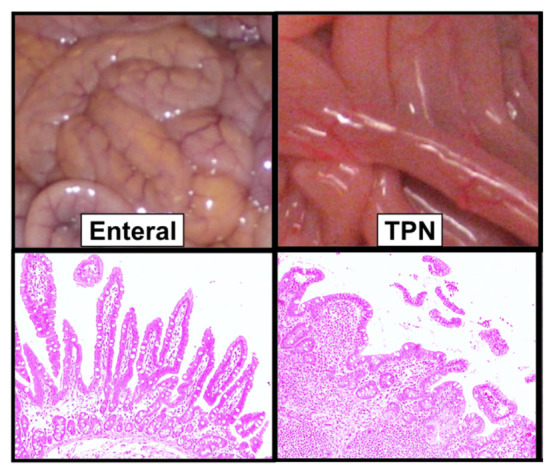
A Macroscopic Appearance and Stained Cross Section of Distal Small Intestine in Preterm Piglets Receiving Enteral Feeding vs. Total Parenteral Nutrition (TPN). Figure modified and reproduced with copyright permission from *American Journal of Physiology-Gastrointestinal and Liver Physiology*, Vol. 302, page 221, Copyright © 2012 by The American Physiological Society. Original article published as “Enteral Bile Acid Treatment Improves Parenteral Nutrition-Related Liver Disease and Intestinal Mucosal Atrophy in Neonatal Pigs” by Jain et al. [52].

However, in utero, the fetal gastrointestinal tract remains in use, with the fetus estimated to swallow amniotic fluid at 200–250 mL/kg/day of weight [12]. In addition to enzymes and electrolytes, amniotic fluid also contains growth hormones, carbohydrates, proteins, and lipids [12]. Therefore, nutrients from the swallowed amniotic fluid support fetal growth, as well as the proliferation of the intestinal epithelial cells. However, preterm birth disrupts this normal physiology, with intestinal development further hindered by late provision of enteral feeding [12]. In animal and human studies, exclusive parenteral nutrition provision increases intestinal villous atrophy—as demonstrated in Figure 2—which is reversed with enteral feeding provision [13,14].

Findings thus support early enteral feeding, with a Cochrane review further concluding no increased risk of NEC in infants born <28 weeks gestation or <1500 g by initiating enteral feeding within the first four days of life [10]. With further consideration to NEC, the American Society for Parenteral and Enteral Nutrition (ASPEN) recommends starting minimal enteral feeding within the first two days of life for infants ≥1000 g [11]. For infants receiving enteral feeding but in need of blood transfusion, a 2019 Cochrane review concluded there is insufficient evidence to indicate if holding enteral feeding has any effect on developing NEC [53]. Similarly, monitoring gastric residuals is not necessary as an assessment of feeding tolerance to prevent NEC, as a 2019 Cochrane review found no benefit to this practice [54]. Continuing enteral feeding also remains feasible and safe for infants receiving Indomethacin as a treatment for patent ductus arteriosus [55,56]. Delayed introduction of feeding, discontinuation of enteral feeding, prolonged trophic feeding, and slow enteral advancement only results in longer duration for achieving full-volume enteral feeding [10,23]. Enteral feeding was previously advanced by 15–20 mL/kg/day, but a 2017 Cochrane review indicates that more rapid advancement by 30–40 mL/kg/day does not increase risk of adverse outcomes in very or extremely low birth weight infants [23].

In combination with preventing intestinal villous atrophy, early enteral feedings in preterm infants also promotes intestinal development. In examples from a piglet model, Hansen et al. compared intestinal outcomes for preterm-born piglets between two groups: group 1 received parenteral nutrition only for the first 5 days of life and group 2 received minimal volume enteral trophic feedings for the first 5 days in addition to parenteral nutrition. Significant results showed that relative to body weight, intestinal weight was higher in the enterally fed group (*p* < 0.001) [57], theorized to be associated with enhanced intestinal development. Similarly, lack of enteral feeding may promote increased permeability of the intestine, which could include pathogenic organisms. In the example from mice, lack of enteral feeding and exclusive parenteral nutrition provision increases risk of bacterial translocation [13]. Clinical evidence from a 2017 Cochrane meta-analysis of very low birth weight infants (*n* = 3753) reported slow enteral advancement to result in “borderline increased risk of invasive infection”, Relative Risk (RR) 1.15 (though 95%Confidence Interval (CI) 1.00–1.32) [23]. Comparatively, Shulman et al. compared intestinal permeability in infants born 26–30 weeks gestation (*n* = 132), in which differences were assessed in infants started on minimal enteral feeding at 4 vs. 15 days of life [15]. Results identified that intestinal permeability was lower at 10 days of life in early-fed infants (*p* < 0.01) [15], supporting the theory that early feeding promotes enhanced intestinal development.

Enteral nutrient provision also promotes the maturation of intestinal function and hormones in infants following preterm birth. For example, research by Berseth and Nordyke assessed intestinal manometry in infants born 26–33 weeks gestation (*n* = 32) receiving enteral feeding during the first week of life of either formula or sterile water (at 24 mL/kg/day) in addition to parenteral nutrition [20]. While both groups showed an initial intestinal motor response to enteral provision, after 10 days infants receiving formula had less clustered phasic activity (*p* < 0.05) and more migrating activity (*p* < 0.01) during fasting states [20]. Furthermore, Berseth compared gastrointestinal hormones and peptides in a small subset of infants (*n* = 27) born at 28–32 weeks gestation with enteral feeding initiated early (3–5 days of life) vs. late (10–14 days of life) [21]. While no differences initially existed, after 10 days, fasting plasma gastrin (*p* = 0.03) and gastric inhibitory peptide (*p* = 0.001) were significantly higher in early fed infants [21]. After another 10–14 days, gastrin levels also remained significantly higher in early fed infants (*p* < 0.02) [21]. More recently, Shanahan et al. analyzed serum intestinal-related hormones in infants born <30 weeks gestation (*n* = 64) [22]. The results showed that serum gastric inhibitory peptide (*p* = 0.001) and peptide tyrosine tyrosine (peptide YY) (*p* = 0.0002) on day of life 7 correlated with percent of nutrition provided enterally, and furthermore with singular enteral fat, carbohydrate, and protein provision (all *p* < 0.001) [22]. Additionally, a multivariate analysis found that peptide YY, glucose-dependent insulinotropic peptide, and leptin levels on day of life 7 were all associated with reduced time to achievement of full enteral feeding (all *p* ≤ 0.05) [22].

### 2.3. Development of the Intestinal Microbiome

The microbiome continually develops throughout the neonatal and pediatric period and many modifiable and non-modifiable factors contribute to its evolution and diversity. One modifiable factor is enteral feeding, which promotes the growth of bacteria in the developing gastrointestinal tract of preterm neonates. In example, Dahlgren et al. compared two groups of infants (total *n* = 47) born >32 weeks gestation during the first month of life: one group received enteral feeding and one group exclusively received total parenteral nutrition. At four weeks of life, the fecal bacterial diversity was significantly reduced in the parenterally fed group (*p* < 0.05) [16]. The impact of enteral nutrition on microbiome development in the preterm infant is complex and not completely understood. Development of the intestinal microbiome in terms of bacterial type, amount, and diversity varies in preterm infants according to type of substrate provided, such as mother’s own milk, heat-processed donor human milk, or formula. However, it is less understood how compositional differences within these substrates impact microbiome development, such as structure of the protein molecules or the type or quantity of fatty acids or prebiotic carbohydrates. Yet, additional and less easily modifiable factors must be considered regarding their impact on microbiome evolution, such as presence of intestinal inflammation or feeding intolerance. Likewise, the microbiome may also vary according to gestational age and postnatal age [58,59,60,61,62,63]. Multiple additional factors may impact the intestinal microbiome, such as mode of delivery or antibiotic use [64,65,66,67].

Consideration of the how the microbiome develops should be of interest to clinicians caring for preterm infants. Research has attempted to quantify if the microbiome of preterm infants who develop NEC is altered compared to healthy controls. A recent meta-analysis reported infants who developed NEC had increased fecal levels of Proteobacteria and decreased quantities of Firmicutes and Bacteroidetes [68]. More prospectively, a review by Stiemsma and Michels summarized the interconnection between early microbiome development and developmental origins of health and disease [69]. Early results from both animal and human studies have linked intestinal dysbiosis with adverse outcomes in later life include asthma, allergic disease, obesity, and neurological or behavioral variations [69]. Ultimately, findings suggest that strategies to optimize the intestinal and microbiota health of these fragile infants may be beneficial. Therefore, in addition to enteral feeding promoting a healthy microbiome, initiation of enteral feeding also poses opportunity to introduce probiotic supplementation. However, as no unanimous recommendations exist for optimal probiotic strain, dose, timing of introduction, or length of duration; supplementation must be evaluated on an individual patient, clinician, or unit level. Risks of supplementation include bacterial translocation or unidentified adverse implications for future health. Alternatively, a 2014 Cochrane review reports a lower risk of severe NEC (>Stage II), RR 0.43, 95% CI 0.33–0.56 (*n* = 5529 infants) and mortality, RR 0.65, 95% CI 0.52–0.81 (*n* = 5112) in preterm or low birth weight infants given probiotics as compared to those who were unsupplemented [70]. For those favoring probiotic supplementation, recommendations have been set forth in a position paper by the European Society for Paediatric Gastroenterology, Hepatology, and Nutrition (ESPGHAN) to guide clinical strategies for probiotic administration and strain selection in preterm infants [71].

### 2.4. Inflammatory Response and Clinical Comorbidities

Fetuses swallow amniotic fluid in utero [12], so preterm birth interrupts this normal physiology. If enteral feeding is not initiated after birth, atrophy of the intestinal villous lining will occur. Further findings identify additional detrimental impacts associated with delayed initiation of enteral feeding after birth. For instance, Konnikova et al. analyzed outcomes for infants born <33 weeks gestation, further comparing early vs. late (< vs. >72 h of life) introduction of enteral feeding after birth [17]. While the late feeding group was born smaller and more critically ill at baseline, they demonstrated higher blood C-Reactive Protein levels (*p* = 0.02) and increased fecal levels of pro-inflammatory interleukin-8 (*p* < 0.05) at two weeks of age. After multivariate analysis (adjusting for baseline characteristics, nutrition, and illness severity), these findings still remained significant. Notably after multivariate analysis, late enteral feeding was associated with a 4.5-fold increase in chronic lung disease with oxygen use at 36 weeks gestation (95% CI 1.8–11.5; *p* = 0.002) and a 2.9-fold increase in any stage of ROP (95% CI 1.1–7.8; *p* = 0.03) [17]. Within a cohort consisting only of extremely low birth weight non-growth-restricted infants, late enteral feeding was associated with a 5.97-fold increase in the odds of developing chronic lung disease (*p* = 0.02) [17]. Overall, infants receiving late enteral feeding were more likely to have two or more comorbidities compared to early fed infants (25 vs. 8%) [17]. Similarly, Wemhöner et al. analyzed infants born <31 weeks gestation and ≤1500 g (*n* = 95) to identify how nutrition differs between infants who developed BPD vs. non-diseased controls [31]. Results found that, while calorie, protein, and carbohydrate intakes were statistically similar during the first two weeks of life, infants who developed BPD received a significantly lower volume of enteral feeding (*p* < 0.04) [31].

### 2.5. Neurodevelopment

Increased nutrition provision in the early weeks of life enhance neurodevelopmental outcomes in preterm infants [2]. However, there may be an advantage to delivering nutrition enterally compared to parenterally. In example, Tottman et al. assessed enteral feeding volume, total fluid, and macronutrient intake during the first one week and one month of life for infants born <30 weeks gestation or <1500 g. Though growth, total macronutrient, and enteral intakes were statistically similar between boys vs. girls (*n* = 478), nutrition-related sex-specific differences were identified [29]. After adjustment for birth gestational age, NEC, and sepsis, increased quartiles of enteral feeding volume in mL/kg/day during the first week (*p* = 0.001) and one month of life (*p* = 0.01) was associated with a higher odds of survival without neuroimpairment in girls (but not boys) [29]. Coviello et al. also compared nutritional intakes in the first four weeks of life for infants born <31 weeks gestation (*n* = 131) with brain volumes assessed by magnetic resonance imaging at term equivalent age [30]. After adjusting for cumulative macronutrient intakes, parenteral nutrition duration, and postnatal growth; increased enteral fat, enteral protein, and enteral calorie provision was each associated with increased brain volumes in the cerebellum and basal ganglia (all *p* < 0.05) [30]. Comparatively, the duration of parenteral nutrition days was inversely associated with volume of the cerebellum, cortical grey matter, basal ganglia, and total brain (all *p* < 0.05), though parenteral macronutrient provision was not assessed [30]. Cognitive outcomes at two years of age were not associated with enteral intake during the first four weeks of life in this study, and there was no comparison between brain volume and developmental outcomes at this timepoint [30].

### 2.6. Barriers to Early Life Enteral Feedings

Table 2 lists barriers to early life enteral feeding practices in preterm infants, with suggested strategies to address these barriers.

One of the most significant barriers to initiating and advancing enteral feeding in preterm infants is clinician fear of NEC. As reviewed previously, multiple systematic reviews and meta-analyses conclude that early enteral feeding initiation and more rapid progression does not increase risk of NEC and, in contrast, instead promotes better immediate and lasting outcomes for the preterm infant population. Therefore, the best strategy to address this barrier is implementation of an evidenced-based enteral feeding protocol within each neonatal care unit [28]. Review of additional clinical practices and protocols should also be assessed by individual neonatal care units that may have secondary effects on enteral feeding, such as use of sedation or paralysis that alter gastrointestinal function and ability to tolerate enteral feeding [74]. These strategies aim to alleviate clinician fear by implementing proven successful feeding strategies, create consistency of enteral feeding practices across different providers, and promote a positive “culture change” within neonatal care [79]. In example from one level III neonatal care unit, initial implementation of an enteral feeding protocol for very low birth weight infants yielded significant improvement in clinical outcomes [72]. This protocol was later modified to parallel newfound evidenced-based practices including enteral feeding initiation within the first day of life, reduction in the trophic feeding period to 48 h in infants born <28 weeks gestation with elimination of the trophic feeding period for infants born >28 weeks gestation, earlier milk fortification (at enteral volumes of 50–60 mL/kg/day) and higher enteral volume initiation and advancement (30–35 mL/kg/day) [24,80]. Clinical implementation found this protocol to be feasible and to result in the faster achievement of full enteral feedings, a decreased need for indwelling central line to deliver parenteral nutrition, and fewer infants with weights plotting <10th% on the Fenton preterm infant growth curve at time of discharge [24].

**Figure 3:**
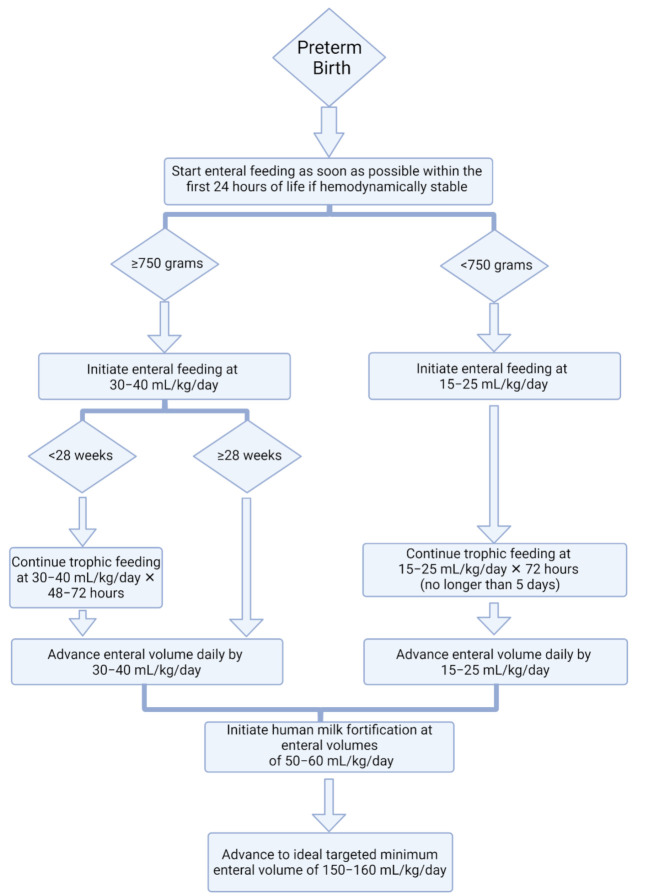
Proposed decision making for initiating and advancing enteral feeding in preterm infants.

A second barrier to initiating and achieving early enteral feeding is determining appropriate methods to use among infants born <750 g, given less available evidence within this population. However, similar strategies and protocols may be implemented as in older or larger preterm populations, but with some modifications, as suggested in Figure 3. For example, enteral volume initiation and advancement may be slower in infants born <750 g, such as with volumes at 15–25 mL/kg/day [81], though some practices may be even slower [26]. Nonetheless, individualized care must be tailored to meet the medical needs of each patient while prioritizing nutrition, especially in those born at the limits of viability.

Concern may exist that the early achievement of full enteral feedings contributes to accelerated growth in this patient population, which contributes to high adiposity. However, risk of excessive growth may be less concerning during this period than growth failure and diagnosable malnutrition [82], as evidenced from 2013 data by the Vermont Oxford Network summarizing that half of very low birth weight infants in North America were discharged with weights plotting <10th% for age on their respective growth chart [83]. Consequently, experienced clinicians may advocate that establishing full volume enteral feeding with appropriate fortification in early life actually supports appropriate body composition by preventing nutrition deficits that cause neonatal growth failure and linear stunting. In example, Miller et al. reported increased risk of growth failure during the transition from parenteral to enteral nutrition [34]. Nutrition management during this transitional period must be carefully evaluated and modified to meet infant needs, especially given the length of this period may vary significantly based on unit nutrition protocols, birth weight and/or gestational age, and infant illness severity. Liotto et al. demonstrated the importance of close observation and monitoring during the transitional phase, as low protein provision during this period was associated with decreased growth velocity and fat-free mass composition at term-corrected age in very low birth weight infants [84]. Likewise, Brennan et al. compared hourly intakes of macronutrients during the transitional phase, exemplifying the need for higher parenteral volume when enteral feeding was <40 mL/kg/day as well as early fortification of human milk to achieve targeted protein provision when infants began receiving more enteral compared to parenteral nutrition [85]. Thus, initiating and achieving targeted fortification levels (that will achieve desired micronutrient, energy, and protein goals at targeted enteral volumes) prior to discontinuation of parenteral nutrition will promote consistent adequate provision of daily nutrition. This may be most beneficial to the smallest preterm infants who consequently have the highest nutritional needs [86], yet may require longer time to transition from parenteral to full enteral nutrition. Enteral feeding fortification is ideally achieved through provision of human milk with designated human milk fortifiers, provision of preterm infant formulas (e.g., 24 calorie/ounce), and additional supplementation (e.g., protein modular or micronutrient supplement) as available and indicated to achieve targeted goals.

**Figure 4:**
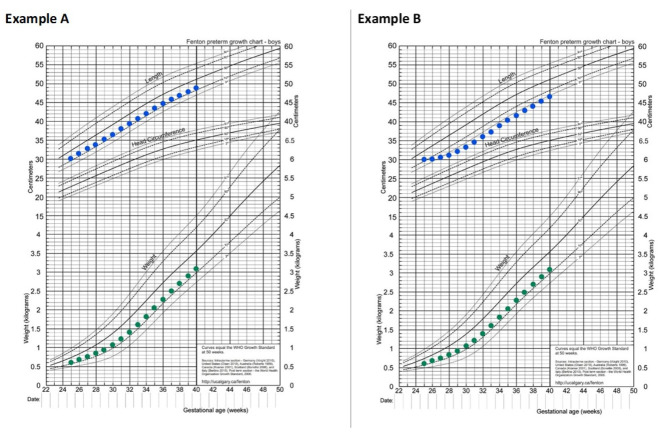
Comparison of adequate (Example **A**) vs. early growth failure (Example **B**) for linear growth in an extremely low birth weight infant from 25–40 weeks gestation with adequate weight gain. Examples A and B 2013 Fenton growth charts constructed and reprinted from the PediTools.org website [89,91,92] as available via open access and under terms of the Creative Commons Attribution License.

**Figure 5:**
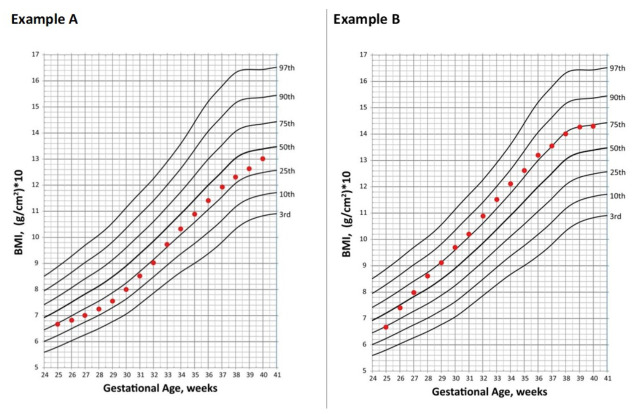
Comparison of body mass index trend from 25–40 weeks gestation in an extremely low birth weight infant with adequate (Example **A**) vs. early growth failure (Example **B**) for linear growth when weight gain is adequate. Examples A and B modified and reproduced with permission from first author Irene Olsen, published in *Pediatrics*, Vol. 135, page 575, Copyright © 2015 by the American Academy of Pediatrics. Original article published as “BMI Curves for Preterm Infants” by Olsen et al. [90].

Clinical experience suggests catch-up growth is more easily attained for weight than for linear growth. Thus, stunted lean body mass accretion in early life may not be regained by term corrected age like weight, and therefore may contribute to a higher proportion of weight as adipose tissue [87,88]. This concept is exemplified in Figure 4 and Figure 5 which were constructed to demonstrate growth from 25–40 weeks gestation for an example infant (birth weight 600 g) on the 2013 Fenton growth curve, with estimated body mass index (BMI) size plotted on the Olsen BMI Curves for Preterm Infants [89,90,91,92]. Figure 4, Example A demonstrates growth appropriately maintained for weight and length, with corresponding BMI plotted in Figure 5, Example A. In contrast, Figure 4, Example B demonstrates the same maintained weight as in Example A, but with linear growth failure in the first one month of life before establishing maintenance. In Figure 5, Example B shows correlating BMI as result of early linear growth failure, which is estimated to plot approximately 0.9 standard deviations higher at 40 weeks gestation compared to if linear growth had been adequately maintained since birth (Example A). While evaluating differences in BMI serves as an indirect estimate of body composition, alternative measures are more accurate, such as air displacement plethysmography [93].

Research additionally indicates that delayed enteral feeding after birth promotes inflammation [17], and inflammation increases risk of both inadequate linear growth and development of comorbidities (e.g., BPD) [94,95]. These may perpetuate the risk of altered body composition and therapies to manage comorbidities (e.g., steroid administration) can further impair linear growth [96]. Conclusively, an early transition to full volume enteral feedings should be viewed as an ideal therapy to promote appropriate growth and body composition in preterm infants. Ultimately, clinicians must consider the comprehensive multi-system short and long-term implications for the management of enteral feeding in preterm infants, especially within the first one month of life.

## 3. Conclusions

Literature supports that enteral feeding, especially early initiation and more rapid enteral advancement, impact preterm infant health during the first one month of life by enhancing micronutrient delivery, promoting intestinal development and maturation, stimulating microbiome development, reducing inflammation, and enhancing brain growth and neurodevelopment. Clinicians must seriously consider the multi-system short and long-term implications that result from the management of enteral feeding in preterm infants and should revise clinical feeding protocols accordingly.

## Figures and Tables

**Table 1:** Benefits and risks of early life enteral feeding practices in preterm infants.

Enteral Feeding Practice	Benefits	Risks
1. Start enteral feeding as soon as feasible following preterm birth [10,11]	○ Use of the gastrointestinal tract is physiologic to the intrauterine environment [12] ○ Prevents villous atrophy and decreases intestinal permeability [13,14,15] ○ Favorably influences intestinal microbiome [16] ○ Reduces risk of developing systemic inflammation [17] ○ Decreases risk of developing comorbidities (e.g., retinopathy of prematurity, bronchopulmonary dysplasia [17]	May need to utilize alternative substrate (e.g., donor human milk or preterm infant formula) to prevent fasting if no maternal milk immediately available [18] As with older populations [19], consider potential implications of enteral feeding in hemodynamically unstable infants
2. Trophic enteral feeding ➔ Brief duration (≤72 h) ➔ Extended duration (>72 h)	○ Promotes neurologic and physiologic gut maturity [20,21,22] ○ Possible decrease in dysmotility of prematurity [20] ○ Brief duration: Decreases time to achieve full volume enteral feedings [23,24] ○ Extended duration: Strategy previously implemented in infants born <750 g to reduce necrotizing enterocolitis risk [25,26], though there are no unanimous recommendations for the most clinically advantageous trophic feeding duration	Brief duration: Less evidence available for application in a population of infants born <750 g or at the limits of viability [23] Extended duration: Inadequate evidence to show clear benefit for infants >750 g [27]
3. Start and advance enteral feeding by 30–40 mL/kg/day to goal volume [23]	○ Evidenced-based practice [23] ○ When fortified, may provide higher micronutrient provision in early life [28] ○ Decreases risk of infection [23] ○ Decreases duration of parenteral nutrition use and need for central line [24] ○ Higher enteral provision enhances brain growth and potential cognitive outcomes [29,30] ○ Higher early enteral provision decreases risk of developing comorbidities [31]	Less evidence available for application in a population of infants born <750 g or at the limits of viability [23]
4. Early human milk fortification [32,33]	○ Increases micronutrient provision [28] ○ Enhances energy and protein provision as parenteral nutrition provision is decreased (a contributing period to suboptimal growth [34])	Increases feeding osmolality [35]

**Table 2:** Barriers and strategies to initiating and advancing early life enteral feeding in preterm infants.

Barriers	Strategies to Address Barriers
1. Fear of necrotizing enterocolitis or spontaneous intestinal perforation	▪ Implementation of an evidenced-based enteral feeding protocol [24,28,72]
2. Dysmotility of prematurity	▪ Early use of suppositories or saline enemas [73] ▪ Decreased use of sedation and paralysis [74]
3. Emesis and/or abdominal fullness [75,76,77,78]	▪ Alternative method of respiratory support than non-invasive ventilation, if able and appropriate [75,76] ▪ Nursing intervention (e.g., pull air out of stomach) [75,76] ▪ Use of continuous drip or every 2 h bolus feedings [77] ▪ Prolong bolus feeding infusion time [77] ▪ Modify body positioning [77] ▪ Use of a hydrolyzed human milk fortifier [77] ▪ Use of transpyloric tube feeding per medical team discretion [78]
4. High gastric residuals	▪ Do not routinely check residuals based on limited evidence to support this practice [54]
5. Hemodynamic instability	▪ Allow feedings with patent ductus arteriosus, even if receiving Indomethacin [55,56] ▪ Develop and implement unit-specific standardized feeding guidelines, including threshold dosing of medication (e.g., vasopressors, etc.) to determine if enteral feedings can be initiated and/or continued [28]
